# Right-Sided Colonic Wall Thickening on Abdominal Computed Tomography as a Potential Adjunctive Imaging Clue in Thrombotic Microangiopathy Presenting With Gastrointestinal Symptoms

**DOI:** 10.7759/cureus.117615

**Published:** 2026-10-07

**Authors:** Koichi Kitamura, Koichi Hayashi, Toshihiko Suzuki

**Affiliations:** 1 Department of Nephrology, Endocrinology, and Diabetes, Tokyobay Urayasu Ichikawa Medical Center, Chiba, JPN; 2 Department of Emergency and Critical Care Medicine, St Marianna University School of Medicine, Kawasaki, JPN

**Keywords:** acquired thrombotic thrombocytopenic purpura (attp), gastrointestinal symptoms, plasma exchange therapy, shiga toxin-producing escherichia coli-associated hemolytic uremic syndrome (stec-hus), stec-hus, thrombotic microangiopathy (tma)

## Abstract

Thrombotic microangiopathy (TMA) presenting with gastrointestinal symptoms poses a diagnostic challenge because Shiga toxin-producing *Escherichia coli*-associated hemolytic uremic syndrome (STEC-HUS) and acquired thrombotic thrombocytopenic purpura (aTTP) share overlapping clinical features but require distinct management.

We report the case of a 22-year-old woman who presented with abdominal pain and hematochezia and subsequently developed TMA. Abdominal computed tomography (CT) demonstrated continuous edematous wall thickening from the terminal ileum and cecum to the ascending colon, raising early suspicion for STEC-HUS. However, a high PLASMIC score prompted urgent empirical plasma exchange, which was unfortunately complicated by anaphylactic shock. Subsequent ADAMTS13 testing revealed preserved activity, excluding severe ADAMTS13 deficiency and strongly arguing against acute aTTP. A clinical diagnosis of probable STEC-HUS was made.

To further assess the diagnostic value of CT, we reviewed eight consecutive TMA cases with gastrointestinal symptoms at our institution. Right-sided colonic wall thickening was present in all five STEC-HUS cases but absent in all three aTTP cases, each of whom demonstrated severe ADAMTS13 deficiency. These preliminary findings suggest that right hemicolon-predominant bowel wall thickening may serve as an early adjunctive imaging clue supporting the diagnosis of STEC-HUS in patients with TMA and gastrointestinal symptoms, rather than a definitive discriminator between STEC-HUS and aTTP.

This index case and institutional review highlight the potential utility of abdominal CT as an adjunctive imaging clue in guiding early diagnostic reasoning in patients with TMA and gastrointestinal manifestations. Validation in larger cohorts with microbiologically confirmed STEC-HUS and ADAMTS13-confirmed aTTP is required to establish the true diagnostic performance of this finding.

## Introduction

Thrombotic microangiopathy (TMA) is a clinical syndrome characterized by thrombocytopenia, microangiopathic hemolytic anemia, and ischemic end-organ injury resulting from platelet-rich microthrombi in the microvasculature [1]. The principal etiologies encompass Shiga toxin-producing *Escherichia coli *(STEC)-associated hemolytic uremic syndrome (HUS), atypical HUS (aHUS), acquired thrombotic thrombocytopenic purpura (aTTP), and secondary TMA. Although these entities share overlapping clinical features, they require fundamentally distinct therapeutic strategies [1,2]. When TMA presents with prominent gastrointestinal manifestations, including abdominal pain, diarrhea, and hematochezia, in a patient without a history of transplantation or malignancy, STEC-HUS is typically the leading diagnostic consideration [3], which is classically preceded by bloody diarrhea following exposure to STEC, most commonly serotype O157:H7 [4]. However, aTTP can also manifest with gastrointestinal symptoms [5], necessitating vigilant differentiation.

The temporal trajectory of establishing a differential diagnosis in TMA is a critical determinant of patient outcomes. Several diagnostic markers and ancillary tests are available for the evaluation of TMA, including Shiga toxin testing, stool culture, ADAMTS13 activity and inhibitor assays, complement activity, clinical prediction scores (i.e., PLASMIC score) [6], and genetic testing for aHUS. The turnaround times for these investigations often range from several days to weeks. This delay creates a substantial diagnostic gap during which clinicians must make urgent management decisions under considerable uncertainty. Indeed, empirical plasma exchange is frequently initiated for presumed aTTP pending confirmatory results, but has limited efficacy in STEC-HUS and is no longer considered first-line therapy for aHUS in the current era of complement inhibition. Moreover, a high PLASMIC score does not reliably distinguish STEC-HUS from aTTP, because severe thrombocytopenia and hemolysis, both hallmarks of STEC-HUS, can yield a high score even in the absence of ADAMTS13 deficiency [7]. Thus, these diagnostic challenges underscore the compelling need for more rapid, accurate, and readily accessible diagnostic modalities.

Radiological imaging not only provides valuable adjunctive information by delineating the anatomical distribution of the disease but may also offer crucial clues to the underlying etiology. Several case reports have described characteristic right-sided colonic wall thickening in STEC-HUS, attributed to submucosal edema in the cecum and ascending colon, regions thought to exhibit heightened susceptibility to Shiga-toxin-mediated endothelial injury [8,9]. However, to date, CT findings in STEC-HUS have not been systematically compared with those observed in aTTP within a single institutional cohort.

The PLASMIC score was developed and validated to predict the probability of severe ADAMTS13 deficiency (<10%) in adults with TMA, but was not specifically designed to differentiate STEC-HUS from aTTP [6]. 

Here, we report a clinically diagnosed case of STEC-HUS in which distinctive CT findings facilitated early diagnostic suspicion. Furthermore, to identify radiological features that may aid in early differentiation, we performed a comparative analysis of five STEC-HUS cases and three cases of aTTP presenting with abdominal symptoms at our institution.

## Case presentation

A 22-year-old Japanese woman with no relevant personal or family medical history and no regular medications presented with acute-onset abdominal pain and hematochezia for five days. She reported no recent travel, antibiotic use, or known contact with individuals with similar illness, although she had eaten sushi several days before symptom onset. Over the preceding days, she developed a prodrome of watery, then bloody, diarrhea and progressive abdominal tenderness before presenting to our institution.

On physical examination, her blood pressure was 157/88 mmHg, temperature 37.8°C, heart rate 88 beats/min, and oxygen saturation 99% on room air. Abdominal examination revealed diffuse tenderness without peritoneal signs, and the neurological examination was unremarkable. Given the acute presentation of abdominal pain and hematochezia without significant fever or peritoneal signs, the initial differential diagnosis included infectious colitis (bacterial, including STEC and other enteropathogens), inflammatory bowel disease, ischemic colitis, and, less likely, a surgical abdomen such as appendicitis. The absence of peritoneal signs and the patient's young age made a surgical emergency less likely, while the preceding watery-to-bloody diarrhea prodrome raised suspicion for an infectious or toxin-mediated colitis. Subsequent laboratory investigations, however, revealed findings consistent with TMA (Table 1), which redirected the differential toward STEC-HUS and aTTP, both of which share overlapping features but require distinct management. A high PLASMIC score (6 points) [6] reflected severe thrombocytopenia, hemolysis, and a modest elevation in serum creatinine in the absence of active malignancy or a history of transplantation. Stool culture was negative for STEC; however, specific Shiga toxin testing, PCR, or serological evaluation was not performed. Total complement activity (CH50) was within normal limits.

**Table 1:** Summary of laboratory investigations at admission

Parameter	Value	Reference range
Hemoglobin	8.6 g/dL	11.5-15.0
Platelet count	29 ×10⁹/L	150-350×10⁹
Lactate dehydrogenase	1268 IU/L	124-222
Creatinine	1.08 mg/dL	0.46-0.79
Haptoglobin	3.0 mg/dL	30-200
C3	101 mg/dL	73-138
C4	27 mg/dL	Nov-31
CH50	51 U/mL	30-53
ADAMTS13 activity	98%	<10% suggests severe deficiency per the International Society on Thrombosis and Haemostasis (ISTH) criteria [7]
ADAMTS13 inhibitor	Negative	Negative
ANA	01:40	<1:40
MPO-ANCA	Negative	Negative
PR3-ANCA	Negative	Negative
Anti-GBM antibody	<1.5 U/mL	<3.0

Renal function at presentation was mildly impaired, with a baseline serum creatinine of 0.70 mg/dL (obtained six months prior to this admission). On admission (symptom day 5), creatinine was 1.08 mg/dL (1.54-fold increase above baseline, Kidney Disease: Improving Global Outcomes Acute Kidney Injury (KDIGO AKI) stage 1) [10], peaking at 1.24 mg/dL on symptom day 7. Urine output was preserved throughout. Urinalysis showed mild hematuria and proteinuria. Creatinine improved to 1.00 mg/dL by discharge (symptom day 17) without renal replacement therapy, consistent with the self-limiting renal course typical of STEC-HUS. 

Contrast-enhanced abdominal computed tomography (CT) performed on symptom day five revealed continuous edematous wall thickening extending from the terminal ileum and cecum to the ascending colon, accompanied by pericolic ascites (Figure 1), a distribution consistent with right hemicolon predominance compatible with STEC-mediated colitis [9,11]. Based on this CT pattern and the preceding episode of bloody diarrhea, STEC-HUS was considered the leading clinical diagnosis. She reported eating sushi several days before symptom onset; however, no epidemiologically confirmed source of STEC exposure was identified, and the high PLASMIC score precluded early exclusion of aTTP. Empirical plasma exchange and high-dose corticosteroids were, therefore, initiated while awaiting confirmatory ADAMTS13 testing.

**Figure 1:**
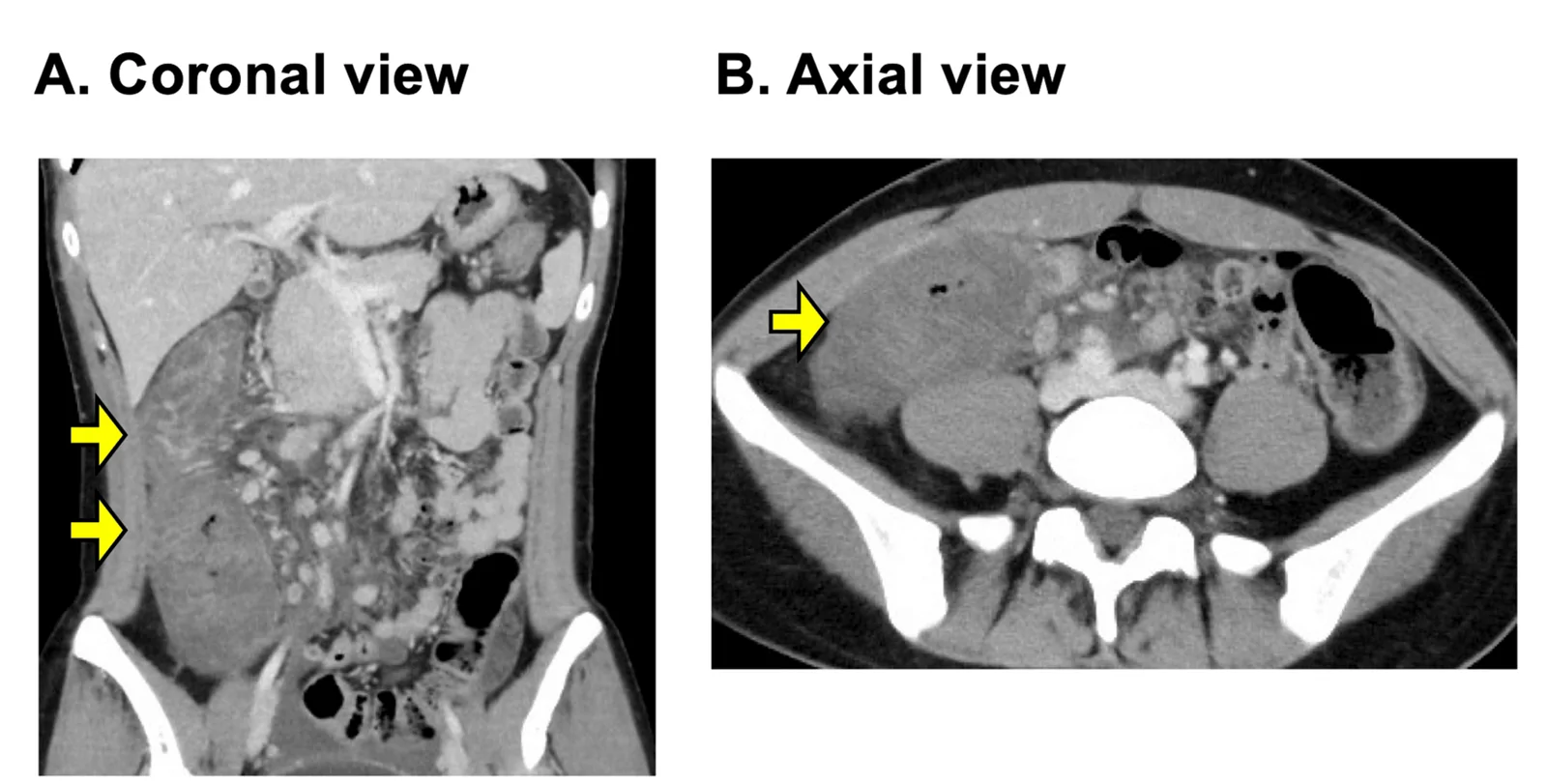
Contrast-enhanced abdominal computed tomography Coronal (A) and axial (B) images, obtained on symptom day five, show continuous edematous wall thickening from the terminal ileum and cecum to the ascending colon, with surrounding ascites (arrows). Yellow arrows denote marked edematous wall thickening of the ascending colon.

The first session of plasma exchange was complicated by anaphylactic shock to fresh frozen plasma, requiring immediate cessation and epinephrine administration, whereas subsequent sessions resulted only in mild urticarial reactions. Platelet count and lactate dehydrogenase subsequently improved. ADAMTS13 activity before plasma exchange was preserved at 98%, which is above the threshold for severe deficiency (<10% per International Society on Thrombosis and Haemostasis (ISTH) criteria [7]), excluding severe ADAMTS13 deficiency. The inhibitor titer was <0.5 BU/mL and was interpreted as negative according to the institutional assay, thereby excluding aTTP and prompting discontinuation of PE. Secondary TMA was excluded by antinuclear antibodies at low titer (ANA 1:40, without clinical significance), negative anti-neutrophil cytoplasmic antibodies, negative anti-glomerular basement membrane antibodies, and the absence of suggestive clinical history. Normal complement levels together with non-pathogenic findings on complement-regulatory gene testing rendered aHUS less likely.

A clinical diagnosis of probable STEC-HUS was established based on the following criteria: (1) a prodrome of bloody diarrhea, (2) characteristic wall thickening of the right hemicolon on CT, (3) exclusion of severe ADAMTS13 deficiency, (4) absence of secondary causes of TMA, (5) normal complement activity, and (6) a self-limiting rapid recovery without complement inhibition. While the diagnostic certainty was limited by the lack of microbiological confirmation of STEC infection, the patient was discharged with improved renal function. Although the admission was complicated by anaphylactic shock during the first plasma exchange session, the patient recovered fully without sequelae.

Methods

We conducted a retrospective review of all patients with TMA presenting with gastrointestinal symptoms documented in the electronic medical records of our institution between January 2014 and December 2023. Cases were identified by searching discharge diagnoses for TMA, HUS, and TTP. CT images were independently assessed by a radiologist and a nephrologist, both blinded to the final diagnosis, for the presence or absence of colonic wall thickening, its distribution (right-sided predominance defined as involvement of the cecum and/or ascending colon), and associated findings (pericolic ascites, mural edema); disagreements were resolved by consensus. STEC-HUS was defined by the presence of: (1) a prodrome of bloody diarrhea, (2) laboratory evidence of TMA, (3) exclusion of severe ADAMTS13 deficiency where measured, (4) exclusion of secondary TMA and aHUS, and (5) a self-limiting clinical course without complement inhibition. Abdominal CT findings were not formal diagnostic criteria but were available to and could influence the treating clinicians' diagnostic impression in some cases, a potential source of incorporation bias addressed in the Discussion. Cases with microbiological confirmation (positive stool culture or Shiga toxin PCR/serology) were classified as confirmed STEC-HUS; those fulfilling clinical criteria without microbiological confirmation were classified as probable STEC-HUS. aTTP was defined by pretreatment ADAMTS13 activity <10%; a detectable inhibitor or anti-ADAMTS13 IgG antibody supported the immune etiology. Of the five STEC-HUS cases, microbiological confirmation by positive stool culture was available in Cases 2, 3, 4, and 5; stool culture yielded *Escherichia coli* O157 in Cases 2, 3, 4, and 5. Case 1 (the index case) had a negative stool culture without Shiga toxin PCR or serological evaluation and is therefore classified as probable STEC-HUS on clinical grounds. ADAMTS13 activity was measured in Cases 1, 2, and 3 (preserved in all three), but was not measured in Cases 4 and 5 during clinical care. We acknowledge that CT findings contributed to the clinical diagnosis of probable STEC-HUS in some cases, representing a potential incorporation bias, which is discussed as a limitation.

## Discussion

This case underscores the diagnostic challenge of distinguishing STEC-HUS from aTTP in patients presenting with TMA and gastrointestinal symptoms. Because confirmatory tests are often delayed, initial management decisions must frequently be made under substantial uncertainty [1]. In our case, empirical plasma exchange was initiated with aTTP considered among the tentative diagnoses; however, the procedure resulted in adverse reactions, including anaphylactic shock [12], thereby highlighting the importance of early recognition of STEC-HUS, a disease for which plasma exchange lacks clear evidence supporting routine use [13]. Alternatively, characteristic colonic swelling observed on abdominal CT (Figure 1) reflects a distinct radiologic feature and underlying pathophysiology of STEC-HUS. Improving early diagnostic assessment to avoid unnecessary continuation of plasma exchange in patients with STEC-HUS therefore remains a clinically relevant goal.

It is important to emphasize that the decision to initiate empirical plasma exchange in our index patient was clinically appropriate and consistent with current guideline recommendations, which advise drawing ADAMTS13 before treatment and initiating plasma exchange plus corticosteroids without waiting for the result when aTTP remains among the differential diagnoses [7,13]. CT findings should not be used to delay or withhold plasma exchange in any patient in whom aTTP cannot be excluded. Rather, the potential role of CT lies in enriching the multimodal diagnostic framework, particularly in cases where clinical features strongly favor STEC-HUS, and in contributing to early diagnostic reasoning alongside clinical and laboratory data.

Notably, our case showed continuous edematous wall thickening from the terminal ileum and cecum to the ascending colon on abdominal CT (Figure 1). This characteristic distribution pattern has been described in several reports of STEC-HUS [9,11]. To further delineate the clinical implications of this predominantly right-sided involvement, we reviewed all patients with abdominal manifestations of TMA, including STEC-HUS and aTTP, documented in the electronic medical records of our institution between 2014 and 2023, as described in the Methods section. We identified eight patients, comprising five cases of STEC-HUS (including the index case) and three of aTTP. The clinical characteristics of all eight institutional cases are summarized in Table 2. Among these patients, right-sided colonic wall thickening was observed in all STEC-HUS cases but was absent in those with aTTP. When interpreted alongside clinical features such as bloody diarrhea, CT findings may provide useful supportive information in the early phase of illness. Although Cases 4 and 5 were elderly patients in whom ADAMTS13 was not measured, both had positive stool cultures confirming STEC infection and exhibited predominant right-sided colonic involvement on CT. In Case 3, subtotal colectomy was performed because of severe and progressive abdominal symptoms that proved refractory to conservative management, rather than because of a specific complication attributable to the CT finding itself.

**Table 2:** Clinical characteristics, laboratory findings, and CT findings in eight patients with TMA manifesting gastrointestinal symptoms at our institution. Abbreviations: TMA, thrombotic microangiopathy; Hb, hemoglobin; Plt, platelet count; LDH, lactate dehydrogenase; PE, plasma exchange; Cr, serum creatinine at admission; Peak Cr, peak serum creatinine during admission; KDIGO, Kidney Disease: Improving Global Outcomes; AKI, acute kidney injury (stage per KDIGO 2012 criteria); STEC-HUS, Shiga toxin-producing *Escherichia coli*-associated hemolytic uremic syndrome; aTTP, acquired thrombotic thrombocytopenic purpura; BU, Bethesda units; NM, not measured; PE, plasma exchange; RTX, rituximab; M, male; F, female For Case 1, baseline serum Cr was 0.70 mg/dL (most recent pre-admission value); admission Cr 1.08 mg/dL represents a 1.54-fold increase (KDIGO AKI stage 1). Cases 4 and 5: ADAMTS13 activity was not measured during clinical care; stool culture was positive for *Escherichia coli* O157 in Cases 2, 3, 4, and 5. All eight patients achieved full renal recovery within one month.

Case	Diagnosis	Age/Sex	Prodrome (days before TMA)	Hb (g/dL)	Plt (×109/L)	LDH (IU/L)	Cr (mg/dL)	Peak Cr (mg/dL)	KDIGO stage	ADAMTS13 activity (%)	Inhibitor (BU/mL)	PLASMIC score	CT abdomen	PE (days)	RTX	Overall Outcome	Renal Outcome
																		1	Probable STEC-HUS (negative stool culture, no Shiga toxin PCR)	22/F	Abdominal pain, hematochezia(five days)	8.6	29	1268	1.08	1.24	1	98	<0.5	6	Right hemicolon wall thickening; ascites	4	—	Full recovery	Full recovery
2	STEC-HUS	26/M	Abdominal pain, hematochezia(six days)	14.8	82	578	1.26	2.01	2	86	<0.5	6	Right hemicolon wall thickening; ascites	0	—	Full recovery	Full recovery																		
																		3	STEC-HUS	77/F	Abdominal pain, hematochezia(six days)	10.4	24	986	0.62	0.86	1	65	<0.5	6	Right hemicolon wall thickening; ascites	0	—	Subtotal colectomy, Full recovery	Full recovery
4	STEC-HUS	85/F	Abdominal pain, hematochezia(seven days)	10.8	3.5	674	1.19	1.19	2	NM	NM	6	Right hemicolon wall thickening	0	—	Full recovery	Full recovery																		
																		5	STEC-HUS	90/F	Hematochezia(four days)	11.1	54	447	3.66	9.79	3	NM	NM	4	Circumferential right hemicolon wall thickening	0	—	Full recovery	Full recovery
6	aTTP	68/F	Altered mental status (two weeks) Abdominal discomfort (two days)	10.9	7	1147	2.12	2.12	3	<1	3.6	6	No bowel wall thickening	6	Yes	Remission (RTX required)	Full recovery																		
																		7	aTTP	68/M	Altered mental status (two days) Abdominal discomfort (two days)	10.4	15	990	2.32	2.32	2	<1	3.4	6	No bowel wall thickening	12	Yes	Remission (RTX required)	Full recovery
																																				8	aTTP	56/M	Altered mental status(two days) Abdominal discomfort (two days)	4.4	14	3364	1.59	2.27	3	<1	3.2	6	No bowel wall thickening	18	Yes	Remission (RTX required)	Full recovery

An important methodological limitation of this retrospective series is the potential for incorporation bias: in Cases 1, 4, and 5, the CT finding of right hemicolon wall thickening contributed to the clinical diagnosis of STEC-HUS, yet the same CT finding is the imaging parameter under evaluation. In Case 1, the diagnosis of probable STEC-HUS rested primarily on clinical criteria including the CT finding, given the negative stool culture. In Cases 4 and 5, CT findings contributed alongside positive stool culture (*Escherichia coli *O157). This circularity means that the observed association between CT pattern and STEC-HUS diagnosis in these cases cannot be used to independently support the diagnostic performance of CT. Prospective studies in larger cohorts with microbiologically confirmed STEC-HUS and ADAMTS13-confirmed aTTP are required to establish the sensitivity, specificity, and positive predictive value of right hemicolon wall thickening in this context.

Several diagnostic pitfalls must be recognized when interpreting abdominal CT findings in patients with suspected TMA. At our institution, no patients with aTTP presenting with gastrointestinal manifestations have exhibited marked edematous wall thickening in any colonic segment (Table 2). In contrast, a previous report described thickening of the ascending and transverse colon in a patient with ischemic colitis secondary to aTTP [14], a finding likely attributable to mesenteric microvascular thrombosis. Although ischemic colitis more commonly involves the splenic flexure and descending colon [15], such an atypical presentation illustrates a potential diagnostic pitfall. This atypical finding, however, derives from a single case report and warrants cautious interpretation. Specifically, colonic wall thickening on CT could be misinterpreted as suggestive of STEC-HUS, thus highlighting the need to integrate imaging features with the clinical context and laboratory data to avoid diagnostic misclassification. 

The mechanism underlying the laterality of colonic involvement in TMA warrants consideration. In STEC-HUS, Shiga toxin directly injures intestinal microvascular endothelial cells through Gb3 binding [8,16], causing localized vascular leakage and marked edema (Figure 2). This injury appears to preferentially affect the right hemicolon, possibly due to its microvascular characteristics and prolonged exposure to toxin-rich luminal contents [16,17]. In contrast, aTTP rarely demonstrates structural bowel abnormalities because its microthrombi formation does not preferentially involve the colonic microcirculation and lacks an enterotoxic mechanism [18]. These findings suggest that prominent edematous thickening of the right-sided hemicolon reflects the biological activity of Shiga toxin, rather than a nonspecific consequence of microthrombi formation inherent to TMA. 

**Figure 2:**
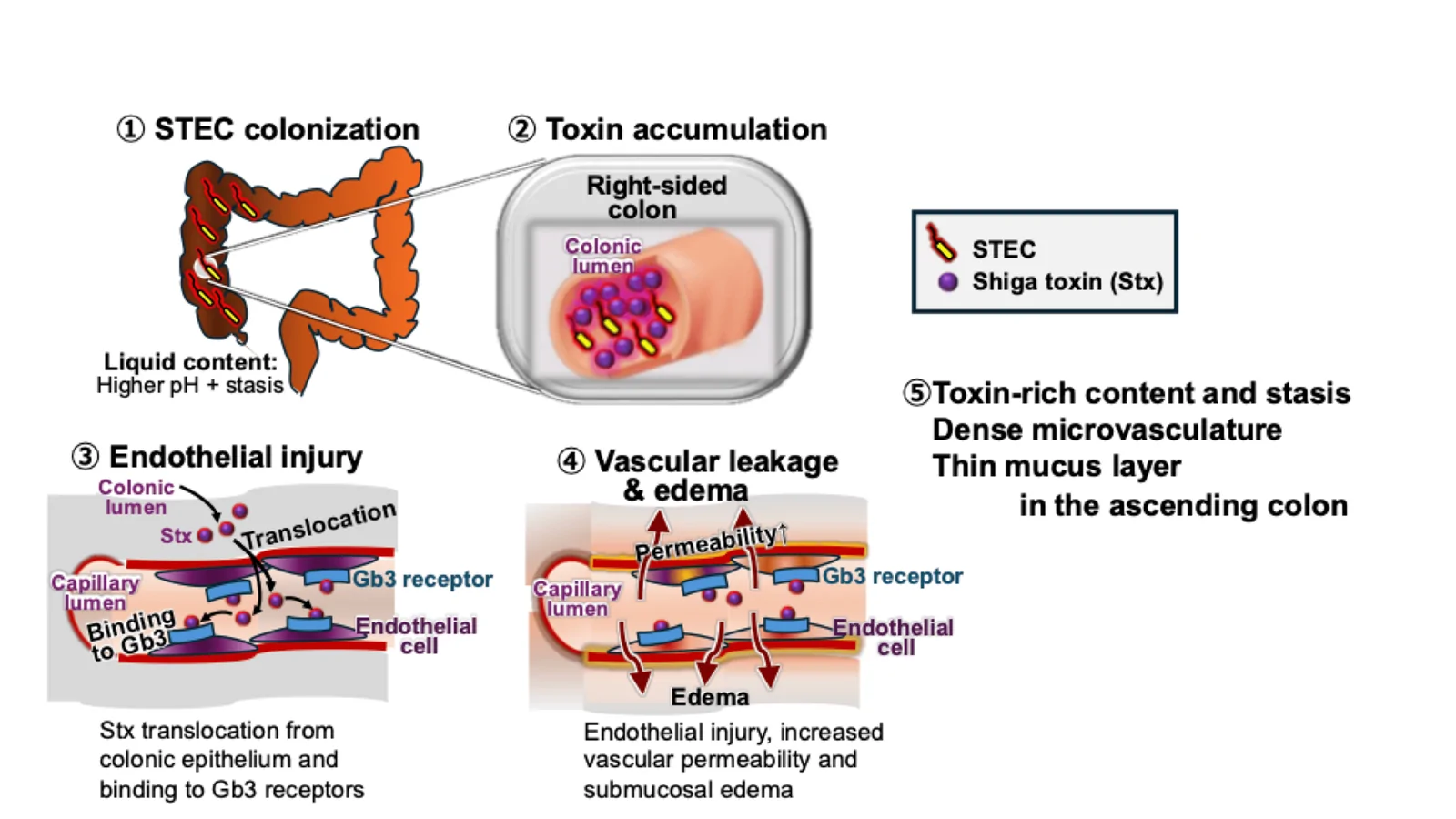
Mechanistic basis for right-sided predominance in STEC-colitis and STEC-HUS Abbreviations: STEC, Shiga toxin-producing *Escherichia coli*-associated hemolytic uremic syndrome; STEC-HUS, Shiga toxin-producing *Escherichia coli*-associated hemolytic uremic syndrome Figure credits: The illustration was created by the authors using Microsoft PowerPoint (Microsoft Corporation, Redmond, WA, USA). An initial conceptual draft was generated with Microsoft Copilot (OpenAI’s GPT-5 (San Francisco, CA, USA)), and the final figure was manually redrawn by the authors. The mechanistic concepts illustrated are based on previously published literature [8,16,17].

Recently, several clinical parameters have been introduced to facilitate the differential diagnosis of TMA. Measurement of ADAMTS13 activity remains the cornerstone for diagnosing aTTP. The turnaround time for obtaining results, however, spans several days despite ongoing efforts to develop rapid assays [19,20]. In our own case series, the PLASMIC score did not contribute to discriminating STEC-HUS from aTTP: all five STEC-HUS patients had scores ranging from 4 to 6 (median = 6 (IQR: 6, 6) calculated using linear interpolation, a range that overlapped completely with that of the three aTTP patients (6 points each). This numerical overlap in our cohort reinforces the previously reported limitation of the PLASMIC score in this setting [6,7] and underscores the need for additional early discriminating tools, such as CT imaging, to support timely clinical decision-making.

## Conclusions

In this small, single-institution retrospective series of eight TMA patients with gastrointestinal symptoms, right hemicolon wall thickening on abdominal CT was observed in all cases clinically diagnosed as STEC-HUS and was absent in all three ADAMTS13-confirmed aTTP cases. These preliminary, hypothesis-generating observations suggest that abdominal CT may offer a useful adjunctive imaging clue to support early diagnostic reasoning in TMA presenting with gastrointestinal symptoms. Abdominal CT findings must be interpreted alongside clinical presentation, microbiological results, and laboratory data, and should not be used to delay or withhold plasma exchange when aTTP cannot be excluded. Prospective studies in larger cohorts with microbiologically confirmed STEC-HUS and ADAMTS13-confirmed aTTP are needed to establish the sensitivity, specificity, and clinical utility of right hemicolon wall thickening as a diagnostic adjunct in this setting.
